# Evidence of Reliable Gastro-Resistance of Novel Enteric Ready-to-Fill Capsules Simplifying Pharmaceutical Manufacturing

**DOI:** 10.3390/pharmaceutics15112592

**Published:** 2023-11-06

**Authors:** Jesús Alberto Afonso Urich, Anna Fedorko, Bettina Hölzer, Johannes Khinast

**Affiliations:** 1 Research Center Pharmaceutical Engineering, Inffeldgasse 13, 8010 Graz, Austria; anna.fedorko@rcpe.at (A.F.); khinast@tugraz.at (J.K.); 2 Institute of Process and Particle Engineering, Graz University of Technology, Inffeldgasse 13, 8010 Graz, Austria; 3 Evonik Operations GmbH, Nutrition & Care, Kirschenallee, 64293 Darmstadt, Germany; bettina.hoelzer@evonik.com

**Keywords:** delayed-release formulation, enteric-coated capsules, targeted drug delivery, acid-labile drugs

## Abstract

Developing delayed-release formulations for acid-sensitive actives can be a costly and time-consuming process. However, ready-to-fill functional capsules, such as EUDRACAP^®^ can significantly mitigate these challenges. The in vitro performance of EUDRACAP^®^ enteric was evaluated in two typical delayed-release scenarios: for diclofenac (a drug that can cause irritation to gastric mucosa), and for omeprazole (a drug susceptible to degradation due to the acidity of gastric fluid). The prototypes were tested in HCl 0.1N according to the USP <711> for at least 2 h and compared to commercial products. The results showed that the performance of EUDRACAP^®^ was below LOD and in compliance with the requirements for drug release in acidic media (NMT 10%). Additionally, the impurities were evaluated after the acidic stress. The low total percentage of impurities of 0.44% for diclofenac (NMT 1.50%) and 0.22% for omeprazole (NMT 2.00%) indicates a very good protection by EUDRACAP^®^. A comprehensive comparative analysis of the in vitro performance clearly showed the acid protection capability of EUDRACAP^®^ enteric capsules making them a serious alternative to existing enteric dosage forms alternatives. EUDRACAP^®^ is an accessible solution both in large-scale industrial and smaller pharmacy settings. Offering increased accessibility, affordability, and convenience to manufacturers and consumers alike and leading to improved healthcare outcomes.

## 1. Introduction

The primary goal of any dosage form is to ensure the accurate and efficient release of a specific amount of drug, resulting in the desired pharmacological response [1].

Hereby, the USP recognizes two main categories of drug release: the immediate release and the modified release. The latter has two further classifications: the delayed release and the extended release [2]. In the context of oral products, such terms as “enteric-coated” and “gastro-resistant” describe the prevention of drug release in the stomach while facilitating it in the intestine [2].

A delayed drug release is typically achieved via enteric coating of dosage forms such as tablets, capsules, and subparts [3]. The primary purpose of enteric coating is to protect the gastric mucosa from potentially irritating drug compounds or to prevent the degradation of the active pharmaceutical ingredient (API) due to acidic conditions or stomach enzymes [4]. 

The majority of film-forming polymers used in enteric coatings are weak acids containing carboxylic acid groups. At low pH, these groups undergo protonation, leaving the polymer non-ionized and insoluble in gastric acid [5]. However, as the pH increases in the small intestine, the carboxylic groups become ionized, resulting in the solubility of the polymer [5], leading to the release of the API. The deprotonation and solubility characteristics of a polymer depend on such factors as the pKa of the acidic groups, the quantity of free carboxylic groups in the polymer chain, and the pH of the surrounding environment. Modifying these properties of the polymer makes it possible to precisely control the release behavior of enteric-coated tablets at specific locations in the small intestine or the colon [6].

**Figure 1:**
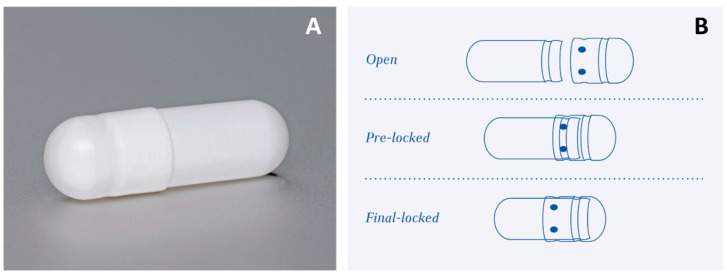
EUDRACAP^®^ enteric capsule (**A**) and opening and closure system (**B**).

Enterically-coated tablets and delayed-release capsules are known for their effective functions. However, this enhanced functionality also adds complexity and cost to the manufacturing process, resulting in higher prices for the drug product. Hence, Evonik has presented as functional pre-locked enteric-protected capsules, denoted as EUDRACAP^®^ enteric (Figure 1). The capsules combine the well-established EUDRAGIT^®^ polymer coating with standard empty pre-locked HPMC capsules [7]. It was specifically designed to accommodate a variety of drug forms, including powders, pellets, granules, and selected liquids. One of the key advantages of these capsules is the easy opening and closing mechanism, which integrates seamlessly with standard capsule filling systems [7].

EUDRACAP^®^ enteric offers robust acid resistance for up to 4 hours without the use of capsule banding [7], ensuring the protection of the encapsulated medication. This concerns the details outlined in the monograph of the Ph.Eur. for capsules [8], where the features of gastro-resistant capsules as delayed-release capsules are described that are intended to resist the gastric fluid and to release their active substance or substances in the intestinal fluid. Additionally, these capsules are designed to be precise in terms of pH targeting, enabling the desired drug release profile. These attributes offer sufficient control of drug delivery. According to the manufacturer, there are additional advantages of this particular product: 

Firstly, the capsules can be manually filled, eliminating the need for specialized equipment and the need for capsule banding [7]. This feature not only simplifies the production process but also opens up possibilities of manufacturing delayed-release dosage forms locally, e.g., in pharmacies. Secondly, the capsules allow direct filling with powder, eliminating the use of coated pellets. This not only streamlines the manufacturing process but also makes it more accessible and cost-effective for consumers.

**Scheme 1:**
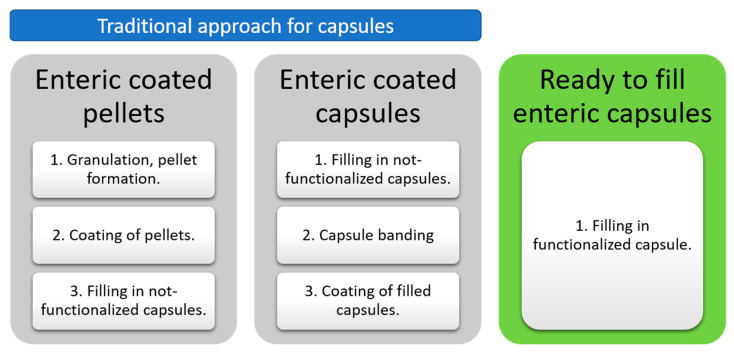
Unit operations for the manufacturing of solid dosage forms in capsules.

The use of pre-locked enteric-protected capsules presents a compelling advantage in terms of manufacturing efficiency in comparison with the traditional approaches (Scheme 1). 

The cases of enteric-coated pellets and enteric-coated capsules demand at least three unit operations, which also imply a meticulous optimization of various process parameters, which can be time- and resource-consuming [9]. However, with a ready-to-fill enteric-protected capsule, the manufacturing process is notably simplified. With only one manufacturing step involved, the need for extensive process development and optimization is significantly reduced. This not only simplifies the production process but also makes it suitable for heat and moisture-sensitive APIs, enhancing versatility and efficiency in pharmaceutical manufacturing. This translates to significant cost savings and acceleration of the drug development time. 

In this paper, the aim was to validate the claims by the manufacturer and to test if indeed the in vitro performance of EUDRACAP^®^ enteric is superior to the existing commercial finished drug products. In order to do so, we carried out a test using two representative drugs that require enteric protection. 

Diclofenac is an example of such a drug with gastric irritant properties [10]. It is a widely used nonsteroidal anti-inflammatory drug for the treatment of chronic arthritis and mild-to-moderate acute pain [11]. It has a short plasma half-life of approximately one to two hours [11]. Various formulations of diclofenac are used for the drug products available in the market or described in the literature [12,13,14,15], including enteric-coated tablets and encapsulated enteric-coated pellets. In this particular case, the capsules are only applied for easier administration and do not have any protective function. As a result, the production is significantly more expensive, because it requires an additional pellet production and coating step before encapsulation. In addition, the protective function of the enteric coating may theoretically decrease, as the surface area exposed to the acidic environment increases.

Also, acid-sensitive drugs, such as proton pump inhibitors, require enteric protection from gastric acid [16]. Omeprazole, a common proton pump inhibitor used for the treatment of gastric acid secretion-related disorders, was introduced in Europe in 1988 and in the United States in the 1990s [17]. Since its introduction, it has played a significant role in the management of patients requiring antisecretory drugs. Omeprazole is known to degrade in acidic environments, depending on the pH levels, while exhibiting acceptable stability under alkaline conditions. Therefore, omeprazole can serve as an example of modified-release delivery [18].

The degradation process of omeprazole is easily detectable since it occurs rapidly and is visually noticeable due to the bright yellow color of the degradation products. Additionally, most of the impurities generated during its degradation are extensively documented in the European and US pharmacopeia [19,20]. In addition, the market offers a wide variety of omeprazole formulations, with enteric-coated tablets and encapsulated pellets being among the most prevalent choices [21,22]. 

In the light above, we selected diclofenac and omeprazole for the evaluation of the enteric protection provided by Evonik’s EUDRACAP^®^ enteric capsules. This evaluation included the assessment of related substances after acidic exposure according to the USP <711> and Ph.Eur. 2.9.3 for delayed-release formulations [23,24] by high-performance liquid chromatography (HPLC), with the goal of comparing their content to an existing marketed finished drug product. 

## 2. Materials and Methods

### 2.1. Reagents and Materials

The solvents used for diluent and mobile phase preparation, were acetonitrile ≥ 99.5% ACS from VWR Chemicals BDH^®^ (Radnor, PA, USA) and methanol HPLC grade methanol ≥ 99.9% from Fisher Chemical™ (Pittsburgh, PA, USA). Mobile phase buffer solutions were prepared with ammonium acetate 99% for HPLC from Loba Chemie PVT. LTD (Mumbai, India) adjusted with acetic acid 100%, Ph.Eur., purchased from Carl Roth GmbH (Karlsruhe, Germany), ammonium bicarbonate Bio Ultra ≥ 99.5% adjusted with ammonia solution 25% both from Sigma-Aldrich (St. Louis, MO, USA). Glycine 99.7–101% reagent Ph. Eur. and Sodium Hydroxide Dri™ ≥ 97% both from Sigma-Aldrich (St. Louis, MO, USA) were used as additional reagents. As dissolution media was prepared 0.1 N solution of HCl 37% from Sigma-Aldrich (St. Louis, MO, USA). Purified water, used for all analyses and sample preparation, was obtained from TKA Germany (Niederelbert, Germany). All samples were filtered with nylon syringe filters for HPLC (0.22 μm) from YETI Merz Brothers GmbH (Haid bei Ansfelden, Austria).

### 2.2. Standards, Samples and Excipients

The pharmaceutical secondary reference standard of diclofenac sodium salt (99.7%) and omeprazole (99.3%) was purchased from Sigma-Aldrich (St. Louis, MO, USA). For capsule filling the same reference standard was used for diclofenac and for omeprazole, it was purchased from Shenzhen Nexconn Pharmatech LTD (Shenzhen, China) (100.0%). The commercial products used as reference were Diclovit^®^ 50 mg gastro-resistant capsules from GL Pharma GmbH (Lannach, Austria) and Losec^®^ 20 mg gastro-resistant capsules from Cheplapharm Arzneimittel GmbH (Greifswald, Germany). The hydroxy naphthol was purchased from Merck (Mumbai, India) and microcrystalline cellulose Vivapur^®^ 200 from JRS Pharma (Mumbai, India). The ready-to-fill EUDRACAP^®^ enteric capsules (size 0) were provided by Evonik Operations GmbH (Essen, Germany). No capsule banding was applied.

### 2.3. Equipment

Dissolution of diclofenac and omeprazole capsules was performed using the dissolution apparatuses Agilent 708-DS (Agilent Technologies, Santa Clara, CA, USA) and Erweka DT 820 (Erweka GmbH, Langen, Germany). HPLC-RP analysis was carried out using Acquity UPLC H-Class^®^ (Waters, Mildorf, MA, USA) and a PDA detector equipped with the chromatographic software Empower 3 (Waters Corp., Milford, CT, USA). The chromatographic columns from Waters Corp. (Milford, CT, USA) used were: Acquity HSS T3 (2.1 × 100 mm; 1.8 µm) for the diclofenac content and impurity analysis; XBridge BEH C18 XP 130A (4.6 × 50 mm; 2.5 µm) for the omeprazole content determination and XBridge C8 Column, 5 µm, 4.6 mm × 150 mm for the determination of omeprazole impurities. pH measurements were performed with the pH-meter FiveEasy from Mettler Toledo (Columbus, OH, USA). For the acid resistance test a disintegration tester DT 1000+ from Labindia Analytical (Navi Mumbai, India) was employed.

### 2.4. Analytical Methodologies

All capsules were placed inside the dissolution vessels with HCl 0.1N for a minimum of two hours, after which they were tested according to the product monographs from the USP, diclofenac sodium delayed-release tablets [25] and omeprazole delayed-release capsules [19]. This corresponds to acid stage testing harmonized on USP <711> and Ph.Eur. 2.9.3 [23,24]. The analytical methods were verified according to the USP <1226> [26] and validated according to the ICH Q2 (R2) [27] and the USP <1225> [28]. 

#### 2.4.1. Diclofenac Determination after Acid Stage Dissolution Test

Six capsules of Diclovit^®^ and EUDRACAP^®^ enteric each containing 50 mg of diclofenac sodium were placed in the dissolution vessels with 900 mL 0.1 N HCl and 50 rpm with paddles at 37 °C [16]. After 120 min, the capsules were taken out of the vessels and transferred to 50 mL volumetric flasks with 1 mL of 5N NaOH. Subsequently, the flasks were filled to approximately 70% capacity with a diluent composed of a 50:50 mixture of water and acetonitrile. The samples were extracted for approximately one hour in total via stirring and ultrasonification. The samples were then made up to volume (1000 µg/mL) for the impurity determinations. For the quantification of content, the samples were diluted to a final concentration of 200 µg/mL. All samples and standards were filtered through a 0.22 µm nylon filter. Furthermore, an additional set of capsules each of Diclovit^®^ and EUDRACAP^®^ enteric were prepared and tested prior to exposure to acid to evaluate their initial content for comparison. The chromatographic methodology conditions are described in Table 1.

#### 2.4.2. Omeprazole Determination after the Acid Stage Dissolution Test

Six capsules of Losec^®^ and EUDRACAP^®^ enteric loaded each with 20 mg of omeprazole were placed in the dissolution vessels with 500 mL 0.1 N HCl and 100 rpm with paddles at 37 °C [20]. After 120 min, the capsules were taken out of the vessels and transferred to 100 mL volumetric flasks with 5 mL of 0.2N NaOH. The flasks were then filled to approximately 70% capacity with a diluent composed of an 80:20 mixture of 10 mM ammonium phosphate buffer pH 8.75 and acetonitrile. The samples were extracted for approximately one hour in total via stirring and ultrasonification. The samples were then made up to volume (200 µg/mL) for the impurity and content determinations. All samples and standards were filtered through a 0.22 µm nylon filter. Also, an additional set of capsules each of Losec^®^ and EUDRACAP^®^ enteric were prepared and tested prior to exposure to acid to evaluate their initial content for comparison. The chromatographic methodology conditions are described in Table 2 and Table 3.

#### 2.4.3. Acid Resistance Test Using Hydroxy Naphthol Blue 

Six capsules of EUDRACAP^®^ enteric loaded with 290 mg of a mixture of hydroxy naphthol blue and MCC (in a ratio of 1:99) were placed in the disintegration tester. The capsules were then individually placed in tubes without discs, and the disintegration process was visually inspected. The disintegration tester was set at a stroke height of 55 ± 1 mm and a stroke frequency of 30 S/min, in 600 mL HCl 0.1N at a temperature of 37 °C. The inspections were performed at 2 and 4 h to determine the disintegration status of all the capsules [8,29]. Samples were taken out of the tubes and carefully dried on the outside with a tissue. After 4 h the capsules were opened and the filling contents were inspected.

## 3. Results

### 3.1. Diclofenac Study Case

Prior to the acid stage testing the samples were analyzed been 99.9 ± 0.2% for EUDRACAP^®^ enteric and 104.3 ± 0.3% for Diclovit^®^. According to the USP, the acceptance criteria for release in the two-hour acid stage is not more than (NMT) 10% [23,24] which can also be interpreted as a drug remaining after the dissolution of not less than (NLT) 90%. For EUDRACAP^®^ enteric 101.6 ± 2.1% were obtained, the difference is not considered to be statistically significant (*p* = 0.2184). On the other hand for Diclovit^®^ after acid exposure 97.0 ± 1.8% were recovered, this difference is considered to be statistically significant (*p* = 0.0003). Both comply with the acceptance criteria proposed by USP, and their difference is considered statistically significant (*p* = 0.0022), according to the unpaired *t* test.

**Figure 2:**
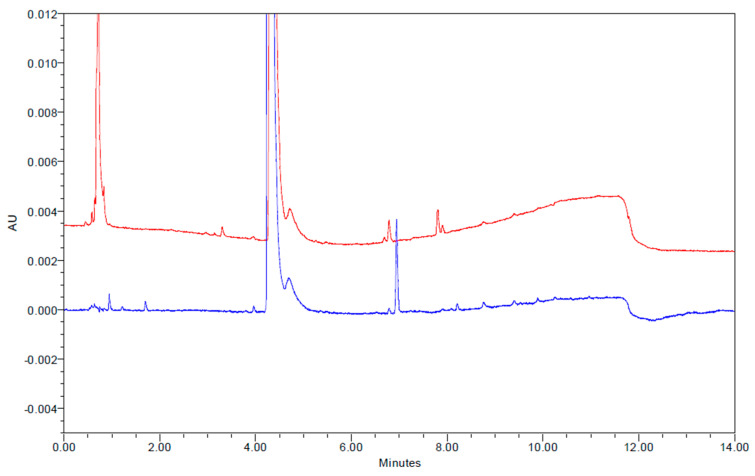
Chromatogram at 280 nm (diclofenac analysis RT 4.3 min) of acid media after 2 h for EU-DRACAP^®^ enteric (blue) and Diclovit^®^ (red).

Regarding the impurities after the acid stage dissolution, the results comply with the acceptance criteria of the USP monograph [16] (see Table 4 and Figure 2).

### 3.2. Omeprazole Study Case

Similarly, the samples were analyzed been 100.5 ± 0.3% for EUDRACAP^®^ enteric and 102.8 ± 2.6% for Losec^®^ prior to acid exposure. The same acceptance criteria [23,24] discussed for the release in the acid stage 101.2 ± 0.79% were obtained for the EUDRACAP^®^ enteric prototype against 98.2 ± 0.95% for Losec^®^. The differences were not statistically different for EUDRACAP^®^ enteric (*p* = 0.2184) but statistically significant for Losec^®^ (*p* = 0.0049). Both comply with the acceptance criteria proposed by the USP, although the difference was statistically significant (*p* = 0.0001) according to the unpaired *t* test. A similar study by Hoelzer and Jain [7] evaluated the release of omeprazole in acidic conditions, reporting a release of below 10% and illustrating the capability of EUDRACAP^®^ enteric in protecting the active ingredient in acidic environments. 

**Figure 3:**
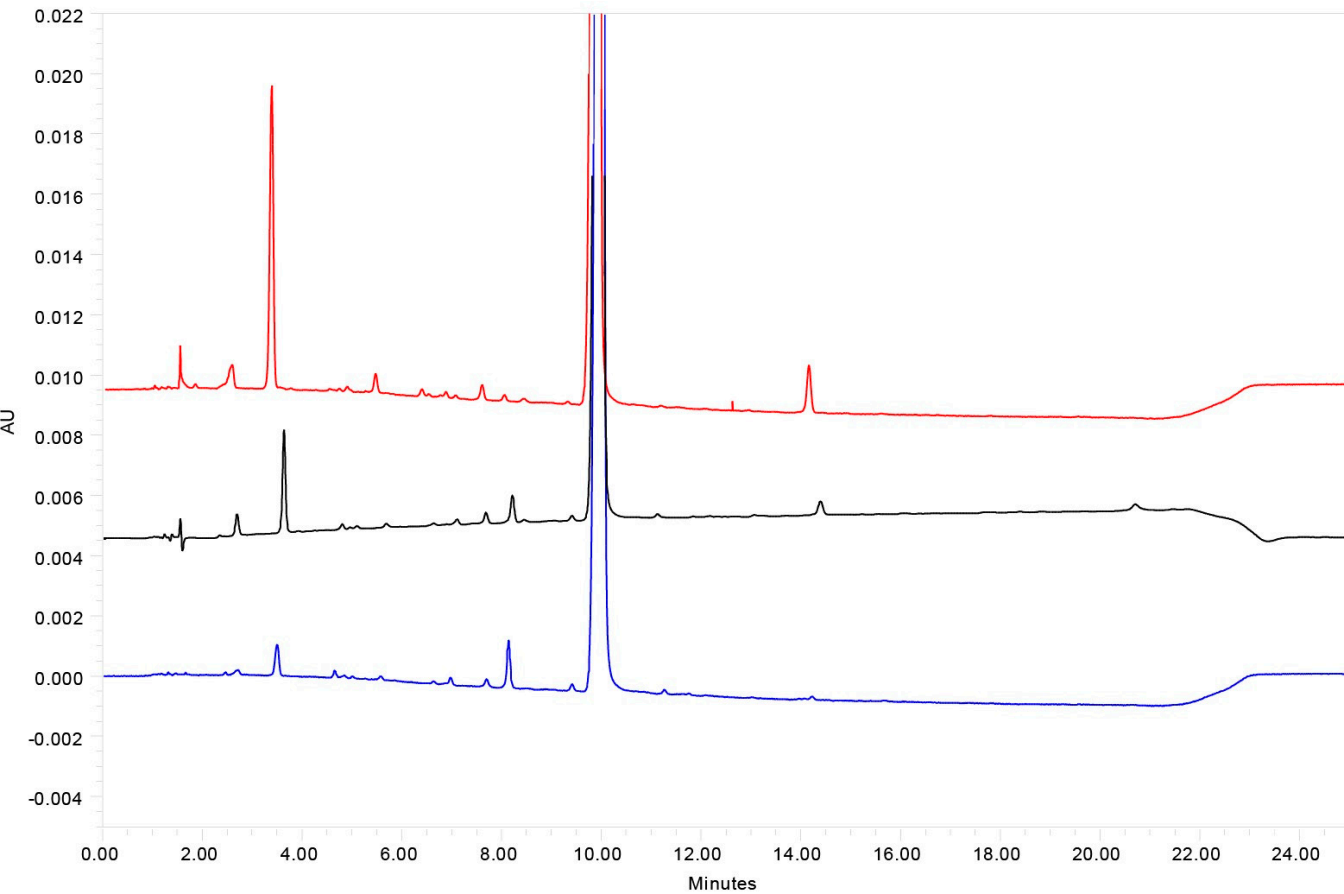
Chromatogram at 305 nm (omeprazole analysis RT 10.8 min) of acid media of EU-DRACAP^®^ enteric after 2 h (blue), 4 h (black) and Losec^®^ after 2 h (red).

Regarding the impurities after the acid stage dissolution, the results comply with the acceptance criteria in the USP monograph [20] (see Table 5 and Figure 3).

### 3.3. Acid Resistance Test

**Figure 4:**
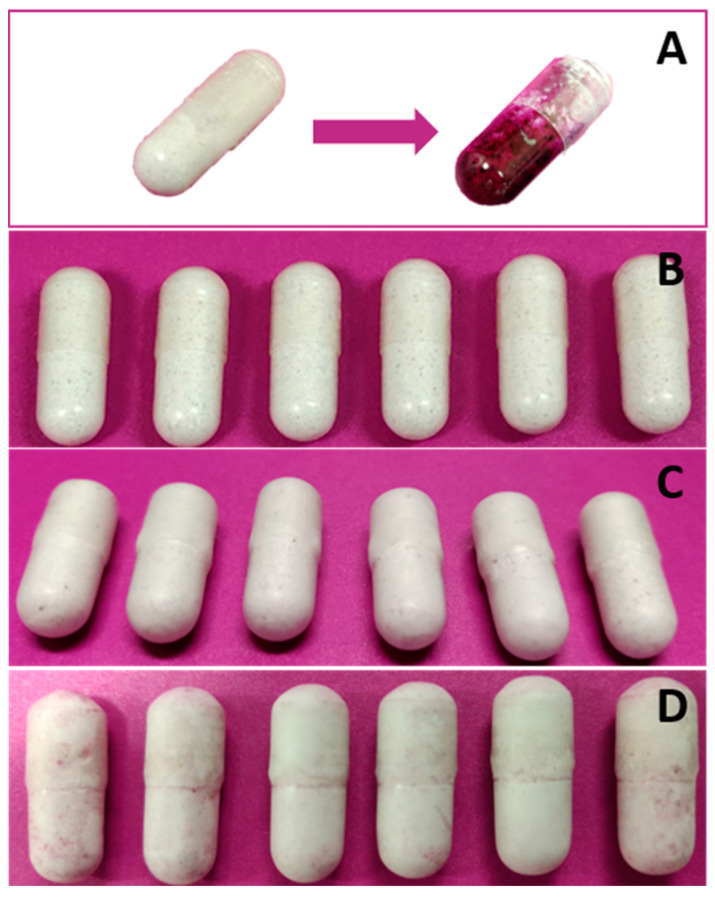
(**A**) Uncoated HPMC capsule before and after adding 2 drops of HCl 0.1 N, (**B**) EU-DRACAP^®^ enteric capsules in the initial stage after filling, (**C**) after 2 h and (**D**) after 4 h of exposure to HCl 0.1 N.

EUDRACAP^®^ enteric capsules were inspected after 2 and 4 h of acid exposure. The protection against acid even after twice the time required by the pharmaceutical regulations is evident [8,23,24,29]. No significant coloring was observed (Figure 4). The consistent results across several studies demonstrate the reliability of EUDRACAP^®^ enteric in pharmaceutical applications.

## 4. Discussion

In the case of diclofenac, the overall level of impurities in each sample was within the acceptable range. Notably, the diclofenac-related compound A, which has to be closely monitored according to the USP monograph [16], was not detected in any of the samples. All of the observed impurities remained within the acceptable range of NMT 0.5%, and the total percentage of impurities was significantly lower than the USP limit, i.e., 0.44% rather than the permitted 1.5% (Table 4). Furthermore, the results align well with the trials conducted for the marketed product Diclovit^®^. 

As for the presence of diclofenac in the acidic dissolution media, in this study, no traces of diclofenac or diclofenac-related compounds were detected in the media after two hours of dissolution during the EUDRACAP^®^ enteric trials (refer to Supplementary Materials) with an LOD/LOQ of 0.10/0.29 µg/mL. These results show the ability of the innovative capsules to protect the gastrointestinal tract from unwanted drug release.

Based on the findings, it can be concluded that, in terms of ensuring patient protection, the in vitro performance of EUDRACAP^®^ enteric capsules is comparable to that of established drug products [10,11].

As mentioned above, omeprazole is known to degrade in acidic environments. Therefore, it becomes very critical for the formulation strategy to protect the drug from acid-induced degradation and ensure the defined targeted release. There was no evidence of free omeprazole, or its degradation compounds present in acid media after two hours, with a LOD/LOQ of 0.05/0.16 µg/mL (see Supplementary Materials).

The analysis shows that the EUDRACAP^®^ enteric prototypes loaded with such a labile drug as omeprazole had all impurities within the acceptable range not only after the required 2 h dissolution period but even after 4 h. This is consistent with the findings from Hoelzer and Jain [7]. The total impurities obtained, as indicated in Table 5, are in line with expectations, with a maximum of 0.22%. Crucially, the standard omeprazole impurities, namely the omeprazole-related compounds F and G, also fall within the acceptable range with a remarkable 0.02%. Surprisingly, the specifically controlled impurity, 5-Methoxy-1H-benzimidazole-2-thiol, specified in the USP monograph, was not detected for the EUDRACAP^®^ enteric prototype.

When compared to Losec^®^, the total impurity level was even lower than that of the marketed product (the total number of impurities of Losec^®^ is 1.51%). Notably, the level of the omeprazole-related compounds F and G in the marketed product slightly exceeded the limit allowed by the USP, measuring at 0.67% compared to the specified 0.5%. Furthermore, it is evident that the levels of impurities in the EUDRACAP^®^ enteric prototype, even after four hours in the acid media, were significantly lower than those in its commercial counterpart (Figure 3).

In addition, with the acid resistance test, hydroxy naphthol blue was selected as a good model for acid or moisture-sensitive capsule fillings. The red color appears upon contact with a small amount of acid, which makes it an effective indicator of acid permeation through EUDRACAP^®^ enteric. No significant acid was observed entering the capsule (Figure 4), but no omeprazole impurities were detected (Figure 3 and Table 5). 

These results demonstrate the protective properties of EUDRACAP^®^ enteric capsules, which effectively protect the compound from degradation in acidic media. The findings show that these capsules maintain their hermetic integrity and prevent the intrusion of surrounding acidic media.

The dissolution properties of EUDRACAP^®^ enteric capsules using omeprazole and caffeine have been already published, proving their compliance with in vitro performance [7]. Before embarking on the expensive and ethically sensitive in vivo testing, comprehensive in vitro evaluations are essential. This includes assessing the impurity formation, disintegration, and dissolution.

Therefore, it is crucial to emphasize that this study was conducted using APIs with a profile like diclofenac and an acid-labile one like omeprazole, serving as models for other sensitive APIs. The results of this in vitro study provide the starting for future investigations involving other types of APIs, such as live biotherapeutic actives and RNA, for example. 

It is well established that pH conditions in the stomach and the small intestine exhibit significant variability, influenced by factors such as individual differences, fasting or fed states [30], stirring pressure by the GI tract [31], and the target absorption profile of the drug [32,33] among others.

The future evaluation of the efficacy of the EUDRACAP^®^ enteric release capsules would greatly benefit from in vivo pharmacokinetic (PK) studies using specific actives.

## 5. Conclusions

In this study, the in vitro performance of EUDRACAP^®^ enteric capsules was examined. The primary objective was to evaluate the capability of these delayed-release capsules to protect the active substance from degradation in the acidic environment of the stomach. As well as protecting the patient from any adverse effects that may result from the premature release of the drug in the gastric environment. To understand these complex dynamics, two drugs were carefully selected that accurately represented the specific scenarios that were investigated.

The ready-to-fill EUDRACAP^®^ enteric capsules fulfilled their fundamental objectives in both experimental scenarios. Their inherent ability to ensure the preservation of the active substance and prevent premature release in the stomach was adequate. This performance result makes EUDRACAP^®^ enteric capsules a serious alternative to existing delayed-release formulations but with the added benefit of simplifying the manufacturing process. This comprehensive comparative in vitro analysis clearly demonstrates the acid protection capability of the novel capsules.

Overall, EUDRACAP^®^ enteric capsules make the production of delayed-release dosage forms easier, not only for large-scale industries but also for smaller pharmacy settings. This option increases accessibility, affordability, and convenience for both manufacturers and consumers, paving the way for improved healthcare outcomes.

## Figures and Tables

**Table 1:** Chromatographic methodology conditions for the diclofenac content and impurities determination.

Chromatographic Feature	Setup
Mobile phases	A: 10 mM ammonium acetate buffer pH 5.3 B: Acetonitrile
Gradient program	%A_0min_ = 70, %A_0.5min_ = 70, %A_8.5min_ = 5, %A_10min_ = 5, %A_10.1min_ = 70 and %A_14min_ = 70
Flow	0.3 mL/min
Injection volume	1 µL
Time per injection	14 min
Column temperature	35 °C
Sample temperature	20 °C
Wavelength	280 nm

**Table 2:** Chromatographic methodology conditions for the omeprazole content determination.

Chromatographic Feature	Setup
Mobile phases	A: 10 mM ammonium bicarbonate buffer pH 8.75 B: Acetonitrile
Gradient program	%A_0min_ = 90, %A_3min_ = 90, %A_10min_ = 40, %A_11min_ = 90 and %A_15min_ = 90
Flow	1.9 mL/min
Injection volume	5 µL
Time per injection	15 min
Column temperature	35 °C
Sample temperature	20 °C
Wavelength	305 nm

**Table 3:** Chromatographic methodology conditions for the omeprazole impurities determination.

Chromatographic Feature	Setup
Mobile phases	A: Glycine 3g/L adjusted to pH 9.0
Gradient program	%A_0min_ = 88, %A_20min_ = 40, %A_21min_ = 88 and %A_25min_ = 88
Flow	1.2 mL/min
Injection volume	10 µL
Time per injection	25 min
Column temperature	20 °C
Sample temperature	20 °C
Wavelength	305 nm

**Table 4:** Diclofenac impurities results for the delayed-release prototypes EUDRACAP^®^ enteric and Diclovit^®^.

Impurity	Acceptance Criteria, NMT (%)	EUDRACAP^®^ NMT (%)	Diclovit^®^, NMT (%)
Oxindole	—	0.00	0.00
Diclofenac-related compound D (diclofenac-bromo analog)	—	0.28	0.28
Diclofenac-related compound A	0.50	0.00	0.00
Diclofenac-alcohol analog	—	0.00	0.03
Diclofenac-benzaldehyde analog	—	0.00	0.01
Any individual unspecified impurity	0.50	0.15	0.28
Total impurities	1.50	0.44 *	0.44 *

* Results mentioned in the table represent just impurities that are under control according to USP, full list of impurities can be found in Supplementary Materials.

**Table 5:** Omeprazole impurity results for the delayed-release prototypes EUDRACAP^®^ enteric and Losec^®^.

Impurity	Acceptance Criteria, NMT (%)	EUDRACAP^®^ after 2 h, NMT (%)	EUDRACAP^®^ after 4 h, NMT (%)	Losec^®^, after 2 h NMT (%)
Omeprazole-related compounds F and G	0.5	0.02	0.22	0.67
5-Methoxy-1H-benzimidazole-2-thiol	0.5	0.00	0.00	0.01
Any other individual impurity	0.5	0.13	0.13	0.29
Total impurities	2	0.22 *	0.74 *	1.51 *

* Results mentioned in the table represent only impurities that are under control according to USP, full list of impurities can be found in Supplementary Materials.

## Data Availability

The data presented in this study are available in supplementary materials.

## Supplementary Materials

The following supporting information can be downloaded at: https://www.mdpi.com/article/10.3390/pharmaceutics15112592/s1, Table S1: Diclofenac impurities summary after acid exposure for EUDRACAP^®^ enteric and Diclovit^®^; Table S2: Diclofenac sodium delayed-release tablets acceptance criteria from the USP; Equation (S1): Percentage of individual impurity calculation for diclofenac sodium delayed-release tablets from USP; Table S3: Omeprazole impurities summary after acid exposure for EUDRACAP^®^ enteric and Losec^®^; Table S4: Omeprazole delayed-release capsules acceptance criteria from the USP; Equation (S2): Percentage of individual impurity calculation for omeprazole delayed-release capsules from the USP; Figure S1: Chromatogram at 280 nm (diclofenac analysis RT 4.3 min) of acid media after 2 h for EUDRACAP^®^ enteric (blue) and Diclovit^®^ (red) and Figure S2: Chromatogram at 305 nm (omeprazole analysis RT 10.8 min) of acid media after 2 h for EUDRACAP^®^ enteric (blue) and Losec^®^ (red).

## Abbreviations

API	Active pharmaceutical ingredient
GI	Gastrointestinal
HCl	Hydrochloric acid
HPLC	High-performance liquid chromatography
HPMC	Hydroxypropyl methylcellulose
ICH	International Council for Harmonization
LOD	Limit of detection
LOQ	Limit of quantification
MCC	Microcrystalline cellulose
NaOH	Sodium hydroxide
NLT	Not less than
NMT	Not more than
Ph.Eur.	European Pharmacopoeia
PK	Pharmacokinetics
RNA	Ribonucleic acid
RT	Retention time
USP	United States Pharmacopoeia
