# Supplementary material for: Evidence of Reliable Gastro-Resistance of Novel Enteric Ready-to-Fill Capsules Simplifying Pharmaceutical Manufacturing
Source: Pharmaceutics. 2023 Nov 6;15(11):2592. doi: 10.3390/pharmaceutics15112592 (PMC10675293; doi:10.3390/pharmaceutics15112592)
Supplement: Supplementary file 1 [file pharmaceutics-15-02592-s001.zip › pharmaceutics-2601715-supplementary.pdf]

pharmaceutics

MDPI

Supplementary Materials: Evidence of Reliable Gastro-Resistance of Novel Enteric Ready-to-Fill Capsules Simplifying Pharmaceutical Manufacturing

Jesús Alberto Afonso Urich, Anna Fedorko, Bettina Hölzer and Johannes Khinast

Table S1. Diclofenac impurities summary after acid exposure for EUDRACAP® enteric and Diclovit®.

Name.	EUDRACAP® after 2h		Diclovit® after 2h	
	Mean %	SD %	Mean %	SD %
Oxindole	0.00	0.00	0.00	0.00
Peak RRT ~0.51	0.01	0.01	0.00	0.00
Peak RRT ~0.76	0.00	0.00	0.02	0.00
Diclofenac-related compound D (diclofenac bromo analog)	0.28	0.02	0.28	0.02
Diclofenac-related compound A	0.00	0.00	0.00	0.00
Diclofenac-alcohol analog	0.00	0.00	0.03	0.00
Peak RRT ~1.60	0.15	0.03	0.04	0.09
Peak RRT ~1.79	0.00	0.00	0.04	0.00
Diclofenac-benzaldehyde analog	0.00	0.00	0.01	0.00
Peak RRT ~2.43	0.00	0.00	0.01	0.02
Total impurities	0.44	0.04	0.44	0.09

Table S2. Diclofenac sodium delayed-release tablets acceptance criteria from USP.

Name	RRT	Acceptance criteria, NMT(%)
Oxindole [a,b]	0.4	—
Diclofenac	1	—
Diclofenac-related compound D (diclofenac bromo analog) [c,d]	1.04	—
Diclofenac-related compound A	1.48	0.5
Diclofenac-alcohol analog [d,b]	1.55	—
Diclofenac-benzaldehyde analog [e,b]	1.81	—
Any individual unspecified impurity	—	0.5
Total impurities [b]	—	1.5

[a] 1,3-Dihydro-2H-indol-2-one. [b] Process-related impurities, not to be counted in total impurities. [c] 2-{2-[(2-Bromo-6-chlorophenyl)amino]phenyl}acetic acid. [d] {2-[(2,6-Dichlorophenyl)amino]phenyl}methanol. [e] 2-[(2,6-Dichlorophenyl)amino]benzaldehyde.

Equation S1. Percentage of individual impurity calculation for diclofenac sodium delayed-release tablets from USP.

$$Result = \left(\frac{rU}{rS}\right) \times \left(\frac{CS}{CU}\right) \times 100$$

rU = peak response of each individual impurity from the Sample solution

rS = peak response of diclofenac from the Standard solution

CS = concentration of diclofenac Sodium in the Standard solution (mg/mL)

CU = nominal concentration of diclofenac sodium in the Sample solution (mg/mL)

Table S3. Omeprazole impurities summary after acid exposure for EUDRACAP® enteric and Losec®.

Name	EUDRACAP® after 2h		EUDRACAP® after 4h		Losec® after 2h	
	Mean %	SD %	Mean %	SD %	Mean %	SD %
Peak RRT ~0.17	0.00	0.00	0.00	0.00	0.06	0.04
Peak RRT ~0.27	0.01	0.01	0.06	0.02	0.24	0.09
Omeprazole-related compounds F and G	0.02	0.05	0.22	0.08	0.67	0.23
Peak RRT ~0.47	0.01	0.01	0.03	0.00	0.00	0.00
Peak RRT ~0.49	0.01	0.01	0.01	0.01	0.02	0.00
Peak RRT ~0.57	0.00	0.00	0.02	0.00	0.07	0.01
5-Methoxy-1H-benzimidazole-2-thiol	0.00	0.00	0.00	0.00	0.01	0.01
Peak RRT ~0.66	0.00	0.00	0.00	0.00	0.01	0.01
Peak RRT ~0.70	0.02	0.00	0.03	0.00	0.02	0.00
Peak RRT ~0.77	0.00	0.00	0.06	0.01	0.05	0.01
Peak RRT ~0.81	0.13	0.02	0.13	0.01	0.03	0.01
Peak RRT ~0.85	0.00	0.00	0.00	0.00	0.03	0.01
Peak RRT ~0.94	0.00	0.00	0.03	0.01	0.00	0.00
Peak RRT ~1.13	0.02	0.00	0.01	0.01	0.01	0.02
Peak RRT ~1.27	0.00	0.00	0.08	0.02	0.00	0.00
Peak RRT ~1.42	0.00	0.00	0.00	0.00	0.29	0.09
Peak RRT ~1.95	0.00	0.00	0.06	0.01	0.00	0.00
Peak RRT ~2.06	0.00	0.00	0.00	0.00	0.00	0.00
Total impurities	0.22	0.06	0.74	0.09	1.51	0.27

Table S4. Omeprazole delayed-release capsules acceptance criteria from the USP.

Name	RRT	RRF	Acceptance criteria, NMT(%)
Omeprazole-related compounds F and G[a]	0.33	1.6	0.5
5-Methoxy-1H-benzimidazole-2-thiol	0.64	3.1	0.5
Any other individual impurity	—	1	0.5
Total impurities	—	—	2

[a] These impurities undergo transformation in the solution to form a conversion product, which elutes at the relative retention time of 0.33.

Equation S2. Percentage of individual impurity calculation for omeprazole delayed-release capsules from the USP.

$$Result = \left(\frac{rU}{rS}\right) \times \left(\frac{CS}{CU}\right) \times \left(\frac{1}{F}\right) \times 100$$

Where:

rU = peak response for each impurity from the Sample solution

rS = peak response for omeprazole from the Standard solution

CS = concentration of omeprazole in the Standard solution (mg/mL)

CU = nominal concentration of omeprazole in the Sample solution (mg/mL)

F = relative response factor

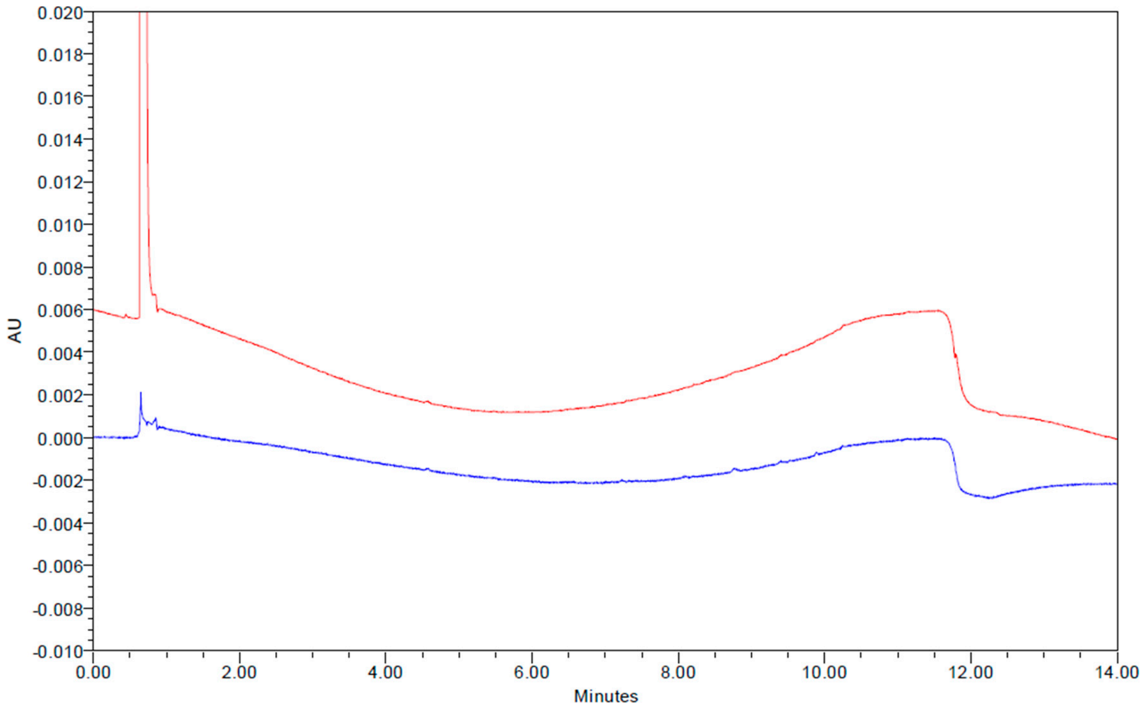

Figure S1. Chromatogram at 280nm (diclofenac analysis RT 4.3 min) of acid media after 2h for EUDRACAP® enteric (blue) and Diclovit® (red).

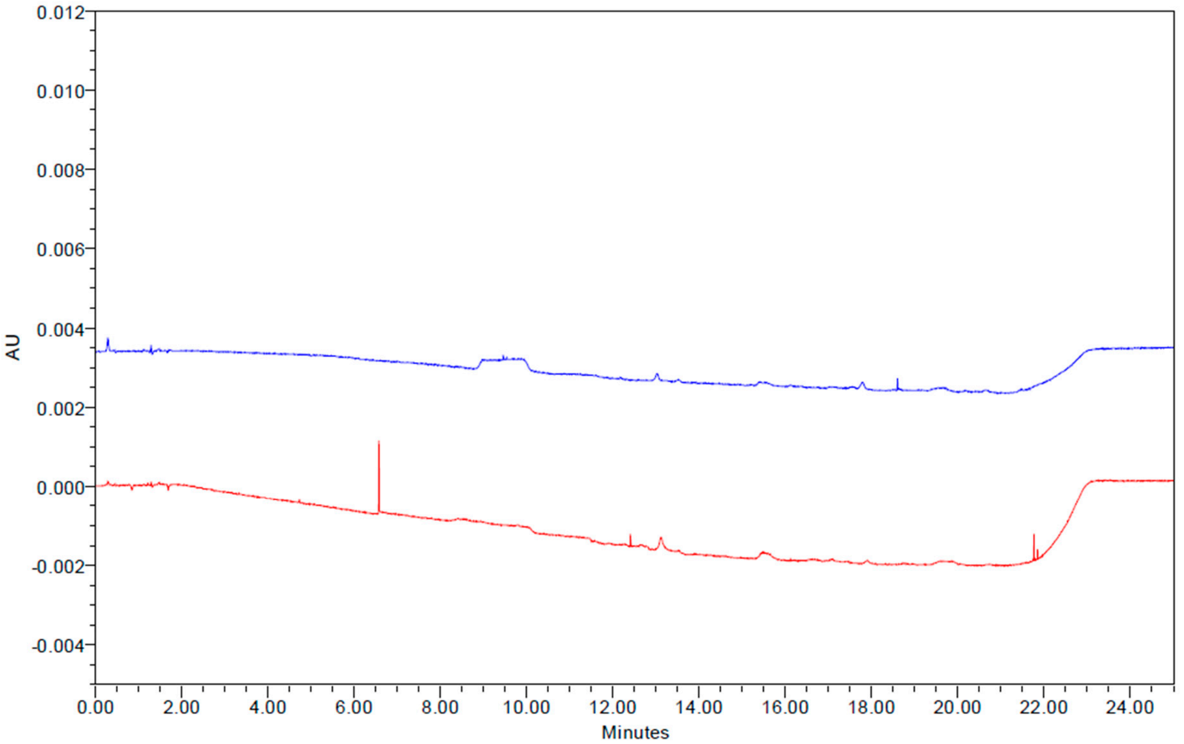

Figure S2. Chromatogram at 305nm (omeprazole analysis RT 10.8 min) of acid media after 2h for EUDRACAP® enteric (blue) and Losec® (red).
